# Longevity-associated mitochondrial DNA 5178 C/A polymorphism modifies effect of aging on renal function in male Japanese health checkup examinees: an exploratory cross-sectional study

**DOI:** 10.1186/s40101-019-0204-3

**Published:** 2019-09-05

**Authors:** Iichiro Ohtsu, Mamoru Ishikawa, Naomi Matsunaga, Kanae Karita, Masao Yoshida, Hirotaka Ochiai, Takako Shirasawa, Takahiko Yoshimoto, Akira Minoura, Shogo Sai, Akatsuki Kokaze

**Affiliations:** 1 Department of Hygiene, Public Health and Preventive Medicine, Showa University School of Medicine, 1-5-8 Hatanodai, Shinagawa-ku, Tokyo, 142-8555 Japan; 2 Department of Public Health, Kyorin University School of Medicine, 6-20-2 Shinkawa, Mitaka-shi, Tokyo, 181-8611 Japan; 3 Mito Red Cross Hospital, 3-12-48 Sannomaru, Mito-shi, Ibaraki, 310-0011 Japan

**Keywords:** Estimated glomerular filtration rate, Kidney function, Longevity, Mitochondrial DNA polymorphism, NADH dehydrogenase, Physiological aging

## Abstract

**Background:**

Mitochondrial DNA 5178 (Mt5178) C/A polymorphism is reportedly associated with longevity in the Japanese population. The objective of this study was to investigate whether Mt5178 C/A polymorphism influences the effect of physiological aging on renal function in male Japanese health checkup examinees.

**Methods:**

A total of 404 male subjects (mean age ± SD, 53.9 ± 7.8 years; range, 29–76 years) were selected from among individuals visiting the hospital for regular medical checkups. After Mt5178 C/A genotyping, a cross-sectional study assessing the joint effects of Mt5178 C/A polymorphism and aging on renal function was then conducted. Renal function was evaluated by estimated glomerular filtration rate (eGFR). Subjects were divided into three age groups (< 50, 50–59, ≥ 60 years).

**Results:**

In simple linear regression analysis, a significant negative association between aging and eGFR was observed in both Mt5178C and Mt5178A genotypic men (*P* < 0.001 and *P* = 0.003, respectively). However, in multiple linear regression analysis, a significant effect of aging on reduced eGFR was observed only in Mt5178C genotypic men (*P* < 0.001). Logistic regression analysis showed that, in the case of reduced eGFR defined as < 75 mL/min/1.73 m^2^, reduced eGFR was dependent on aging in both Mt5178C and Mt5178A genotypic men (*P* for trend < 0.001 and *P* for trend = 0.002, respectively). After adjusting for smoking status and alcohol consumption, reduced eGFR was also dependent on aging in both Mt5178C and Mt5178A genotypic men (*P* for trend < 0.001 and *P* for trend = 0.014, respectively). However, in reduced eGFR defined as < 90 mL/min/1.73 m^2^, reduced eGFR was dependent on aging only in Mt5178C genotypic men (*P* for trend < 0.001).

**Conclusions:**

This cross-sectional study suggests that Mt5178 C/A polymorphism modulates the effects of physiological aging on kidney function in Japanese men.

## Background

Physiological aging, even in healthy aging, is associated with changes in kidney function, for example, glomerular filtration rate (GFR), sodium resorption, transtubular potassium gradient, urinary concentration, renal vascular resistance, and plasma flow [1–3]. Among these physiological changes, a decline in GFR with normal aging is widely known [1, 2].

The mitochondria play a key role not only in aging [4] but also in inherited and acquired kidney diseases [5]. Higher mitochondrial DNA copy number in peripheral blood is associated with a lower risk of incident chronic kidney disease (CKD) [6]. Moreover, several mitochondrial DNA polymorphisms are reportedly associated with renal diseases [7, 8]. Mitochondrial DNA 5178 (Mt5178) C/A polymorphism, which is also recognized as NADH dehydrogenase subunit-2 237 (ND2-237) Leu/Met polymorphism, is originally reported to be associated with longevity in the Japanese population [9]. The frequency of Mt5178A genotype is significantly higher in Japanese centenarians than in the general population [9]. Several clinical epidemiological studies reported that Japanese individuals with Mt5178A genotype are more resistant to adult-onset diseases than those with Mt5178C genotype [10–14]. Moreover, our previous research reported that Mt5178 C/A polymorphism modulates the effect of alcohol consumption [15] or green tea consumption [16] on renal function, evaluated by estimated GFR (eGFR). Although an association between Mt5178 C/A polymorphism and physiological aging effect on pulmonary function was reported [17], an association between Mt5178 C/A polymorphism and effect of physiological aging on kidney function has not yet been reported.

The objective of this exploratory cross-sectional study is to investigate whether Mt5178 C/A polymorphism modifies the effect of physiological aging on renal function.

## Subjects and methods

### Study subjects

Study subjects were recruited from among individuals visiting the Mito Red Cross Hospital for regular medical checkups between August 1999 and August 2000. This study was conducted in accordance with the Declaration of Helsinki. The Ethics Committee of Kyorin University School of Medicine approved the study protocol. Written informed consent was obtained from 602 volunteers before participation. Because of the insufficient number of women available for classification into groups based on Mt5178 C/A genotype and age, female health checkup examinees were excluded. Diabetic patients undergoing treatment were also excluded. Moreover, male health checkup examinees with unclear data were excluded. Finally, 404 Japanese men (mean age ± SD, 53.9 ± 7.8 years; range, 29–76 years) were included in the analysis.

### Anthropometric and clinical characteristics of subjects

Subjects’ data on age, height, weight, blood pressure, serum lipid level, fasting plasma glucose level, serum uric acid level, blood urea nitrogen (BUN) level, and serum creatinine level were collected from the results of regular medical checkups. Renal function was evaluated by eGFR. The eGFR value was calculated using a three-variable Japanese equation: eGFR = 194 × creatinine^−1.094^ × age^−0.287^ [18]. Body mass index (BMI), which is reported to be associated with eGFR [19], was defined as the ratio of subject weight (kg) to the square of subject height (m^2^). Information on antihypertensive medication, which is reportedly associated with kidney function [20], was derived from the subjects’ health records. For antihypertensive medication use, subjects were classified as taking no drug treatment or taking medicine. A survey of habitual smoking, alcohol consumption, and green tea consumption was performed by means of a questionnaire. Smoking status was classified based on the number of cigarettes smoked per day (never- or ex-smokers, 1–20 cigarettes smoked per day, and ≥ 21 cigarettes smoked per day). Alcohol consumption was classified based on drinking frequency (non- or ex-drinkers; occasional drinkers, which included those who drink several times per week or per month; and daily drinkers). Green tea consumption was classified based on the number of cups of green tea per day (≤ 1 cup per day, 2–3 cups per day, 4–5 cups per day, and ≥ 6 cups per day). Selection of these lifestyle habits was based on previous research [15, 16, 21].

### Genotyping

Mt5178 genotyping methods have been described previously [22]. Briefly, DNA was extracted from white blood cells. Mt5178 C/A genotype was determined using the polymerase chain reaction-restriction fragment length polymorphism test. The absence or presence of an *Alu*I site was designated as Mt5178A or Mt5178C, respectively.

### Statistical analyses

All statistical analyses were performed using SAS statistical software (version 9.4 for Windows; SAS Institute, Inc., Cary, NC, USA). Based on a large-scale cross-sectional study of CKD in Japanese adults undergoing an annual health checkup [23], subjects were divided into three age groups (< 50, 50–59, and ≥ 60 years). Multiple logistic regression analysis was conducted to calculate odds ratios (ORs) for the risk of reduced eGFR. Reduced eGFR was defined as < 90 mL/min/1.73 m^2^ [15, 16, 24–26] or < 75 mL/min/1.73 m^2^ [27]. For simple and multiple linear regression analyses, age (< 50 years = 1, 50–59 years = 2, ≥ 60 years = 3), habitual smoking (never- or ex-smokers = 0, 1–20 cigarettes smoked per day = 1, ≥ 21 cigarettes smoked per day = 2), alcohol consumption (non- or ex-drinkers = 0, occasional drinkers = 1, daily drinkers = 2), green tea consumption (≤ 1 cup per day = 1, 2–3 cups per day = 2, 4–5 cups per day = 3, ≥ 6 cups per day = 4), and antihypertensive treatment (not receiving antihypertensive treatment = 0, receiving antihypertensive treatment = 1) were numerically coded. Multicollinearity of independent variables was statistically tested using variance inflation factor values. For the multiple logistic regression analysis, the abovementioned numerical codes were also used. In the final multiple logistic regression model, only independent variables that reached *P* < 0.05 in the simple linear regression model were included. Differences with *P* values < 0.05 were considered to be statistically significant.

## Results

No significant differences in anthropometric or clinical characteristics, including BUN levels, serum creatinine levels, or eGFR, were observed between Mt5178C and Mt5178A genotypes (Table 1). The prevalence of eGFR of < 90 mL/min/1.73 m^2^ was 82.4% in Mt5178C genotypic men and 75.2% in Mt5178A genotypic men, prevalence of eGFR of < 75 mL/min/1.73 m^2^ was 40.5% in Mt5178C genotypic men and 33.2% in Mt5178A genotypic men. The prevalence of moderately decreased eGFR of < 60 mL/min/1.73 m^2^, generally defined as CKD, was 4.5% in Mt5178C genotypic men and 2.6% in Mt5178A genotypic men. The chi-squared test indicated no significant differences in renal function evaluated by eGFR. Furthermore, there were no significant differences in antihypertensive medications, habitual smoking, alcohol consumption, or green tea consumption between Mt5178 C/A genotypes.

**Table 1:** Anthropometric and clinical characteristics of study subjects by Mt5178 C/A genotype

	Mt5178C, *N* = 247	Mt5178A, *N* = 157	*P* value
Age (years)*	54.4 (7.8)	53.2 (7.8)	0.142
Body mass index (kg/m^2^)*	23.3 (2.8)	23.5 (2.6)	0.366
Systolic blood pressure (mmHg)*	125.8 (15.8)	125.7 (14.1)	0.934
Diastolic blood pressure (mmHg)**	73.9 (10.6)	73.7 (9.1)	0.817
LDL cholesterol (mg/dL)*	121.3 (34.4)	118.0 (30.8)	0.319
HDL cholesterol (mg/dL)**	54.5 (13.5)	56.2 (16.1)	0.285
Triglyceride (mg/dL)*	137.4 (91.2)	139.4 (90.2)	0.826
Fasting plasma glucose (mg/dL)**	99.7 (18.9)	98.2 (11.0)	0.334
Uric acid (mg/dL)*	5.94 (1.24)	5.94 (1.22)	0.970
Blood urea nitrogen (mg/dL)*	15.8 (3.7)	15.3 (3.4)	0.157
Creatinine (mg/dL)*	0.813 (0.118)	0.798 (0.111)	0.187
Estimated glomerular filtration rate (eGFR; mL/min/1.73 m^2^)*	79.5 (13.6)	81.4 (12.4)	0.157
Renal function (≥ 90/75–90/60–75/< 60) (%)***	18.6/40.9/36.0/4.5	24.8/42.0/30.6/2.6	0.311
Smoking status (never- or ex-smoker/1–20 cigarettes per day/≥ 21 cigarettes per day) (%)***	59.1/29.2/11.7	59.2/25.5/15.3	0.500
Alcohol consumption (non- or ex-drinker/occasionally/daily) (%)***	18.2/35.2/46.6	13.4/38.2/48.4	0.431
Green tea consumption (≤ 1 cup per day/2–3 cups per day/4–5 cups per day/≥ 6 cups per day) (%)***	26.3/30.3/21.9/21.5	28.0/33.8/17.7/18.5	0.782
Antihypertensive medication (%)***	19.4	13.4	0.115

Age, body mass index, systolic blood pressure, diastolic blood pressure, LDL cholesterol levels, HDL cholesterol levels, triglyceride levels, fasting plasma glucose levels, uric acid levels, blood urea nitrogen levels, creatinine levels, and eGFR are given as means (SD). All *P* values depict significant differences between Mt5178C and Mt5178A genotypic men

*Student’s *t* test, **Welch’s *t* test, ***Chi-squared test

In simple linear regression analysis, age was significantly and positively associated with BUN level in both Mt5178C and Mt5178A genotypic men (*P* = 0.015 and *P* < 0.001, respectively) (Table 2). Alcohol consumption was significantly and negatively associated with serum creatinine level only in Mt5178A genotypic men (*P* = 0.048). Age was significantly and negatively associated with eGFR in both Mt5178C and Mt5178A genotypic men (*P* < 0.001 and *P* = 0.003, respectively). Smoking status and alcohol consumption were significantly and positively associated with eGFR only in Mt5178A genotypic men (*P* = 0.043 and *P* = 0.004, respectively). Multiple linear regression analysis indicated that variance inflation factor values showed low collinearity for all independent variables. In multiple linear regression analysis, age was also significantly and positively associated with BUN level in both Mt5178C and Mt5178A genotypic men (*P* = 0.038 and *P* < 0.001, respectively). Age was significantly and negatively associated with eGFR only in Mt5178C genotypic men (*P* < 0.001). Alcohol consumption was significantly and positively associated with eGFR only in Mt5178A genotypic men (*P* = 0.010).

**Table 2:** Simple and multiple linear regression models for blood urea nitrogen, creatinine, and estimated glomerular filtration rate by Mt5178 C/A genotype

Simple linear regression model										
	Blood urea nitrogen			Creatinine			Estimated glomerular filtration rate			
	Regression coefficient	Standard error	*P* value	Regression coefficient	Standard error	*P* value	Regression coefficient	Standard error	*P* value	
Mt5178C										
Age	0.806	0.329	0.015	0.018	0.010	0.087	− 5.900	1.159	< 0.001	
Body mass index	− 0.084	0.085	0.324	0.003	0.003	0.241	− 0.274	0.312	0.381	
Smoking status	− 0.623	0.338	0.067	− 0.004	0.011	0.705	1.113	1.244	0.372	
Alcohol consumption	− 0.029	0.315	0.925	− 0.004	0.010	0.705	0.791	1.150	0.492	
Green tea consumption	0.216	0.217	0.320	0.004	0.007	0.546	− 1.281	0.790	0.106	
Antihypertensive medication	− 0.313	0.598	0.601	0.015	0.019	0.433	− 2.565	2.186	0.242	
Mt5178A										
Age	1.229	0.342	< 0.001	− 0.001	0.012	0.956	− 3.877	1.273	0.003	
Body mass index	0.034	0.103	0.745	0.002	0.003	0.549	− 0.224	0.379	0.555	
Smoking status	− 0.582	0.360	0.109	− 0.021	0.012	0.075	2.694	1.320	0.043	
Alcohol consumption	− 0.180	0.383	0.639	− 0.025	0.012	0.048	3.996	1.376	0.004	
Green tea consumption	− 0.014	0.253	0.957	0.007	0.008	0.400	− 1.798	0.922	0.053	
Antihypertensive medication	0.146	0.793	0.855	0.041	0.026	0.117	− 5.445	2.887	0.061	
Multiple linear regression model										
	Blood urea nitrogen			Creatinine			Estimated glomerular filtration rate			
	Partial regression coefficient	Standard error	*P* value	Partial regression coefficient	Standard error	*P* value	Partial regression coefficient	Standard error	*P* value	Variance inflation factor
Mt5178C										
Age	0.722	0.346	0.038	0.018	0.011	0.109	− 5.811	1.226	< 0.001	1.108
Body mass index	− 0.085	0.086	0.321	0.004	0.003	0.206	− 0.368	0.304	0.228	1.034
Smoking habit	− 0.612	0.346	0.079	0.001	0.011	0.951	0.036	1.226	0.976	1.059
Alcohol consumption	0.056	0.321	0.863	− 0.007	0.010	0.528	1.126	1.138	0.323	1.069
Green tea consumption	0.136	0.222	0.542	0.000	0.004	0.951	− 0.296	0.786	0.707	1.073
Antihypertensive medication	− 0.733	0.623	0.241	0.010	0.020	0.615	− 0.916	2.206	0.678	1.109
Mt5178A										
Age	1.370	0.375	< 0.001	− 0.012	0.013	0.308	− 2.653	1.359	0.053	1.194
Body mass index	0.052	0.101	0.605	0.002	0.003	0.596	− 0.284	0.365	0.438	1.027
Smoking habit	− 0.414	0.357	0.248	− 0.020	0.012	0.096	1.863	1.293	0.152	1.036
Alcohol consumption	− 0.126	0.378	0.739	− 0.025	0.013	0.052	3.580	1.369	0.010	1.042
Green tea consumption	− 0.306	0.259	0.240	0.005	0.009	0.603	− 0.679	0.939	0.471	1.122
Antihypertensive medication	− 0.756	0.805	0.349	0.043	0.027	0.115	− 3.146	2.916	0.282	1.106

Variance inflation factor shows the value when all covariates are in the multiple linear regression model

In the case of reduced eGFR defined as < 90 mL/min/1.73 m^2^, for subjects with Mt5178C genotype, reduced eGFR was dependent on aging (*P* for trend < 0.001) (Table 3). The OR for reduced eGFR was significantly higher in those aged 50–59 and ≥ 60 years than in those aged < 50 years (OR = 3.100, 95% confidence interval (CI) 1.509–6.370, *P* = 0.002 and OR = 5.007, 95% CI 1.863–13.45, *P* = 0.001, respectively). After adjusting for smoking status and alcohol consumption, reduced eGFR was dependent on aging (*P* for trend = 0.001). The adjusted OR for reduced eGFR was significantly higher in those aged 50–59 and ≥ 60 years than in those aged < 50 years (adjusted OR = 3.086, 95% CI 1.497–6.362, *P* = 0.002 and adjusted OR = 4.206, 95% CI 1.506–11.75, *P* = 0.006, respectively). In contrast, for subjects with Mt5178A genotype, no significant statistical association between aging and reduced eGFR was observed. In the case of reduced eGFR defined as < 75 mL/min/1.73 m^2^, reduced eGFR was dependent on aging in Mt5178C and Mt5178A genotypic men (*P* for trend < 0.001 and *P* for trend = 0.002, respectively). For subjects with Mt5178C genotype, the OR for reduced eGFR was significantly higher in those aged 50–59 and ≥ 60 years than in those aged < 50 years (OR = 3.937, 95% CI 1.832–8.461, *P* < 0.001 and OR = 8.391, 95% CI 3.596–19.58, *P* < 0.001, respectively). For those with Mt5178A genotype, the OR for reduced eGFR was significantly higher only in those aged ≥ 60 years than in those aged < 50 years (OR = 4.343, 95% CI 1.733–10.89, *P* = 0.002). After adjusting for smoking status and alcohol consumption, in both Mt5178C and Mt5178A genotypic men, reduced eGFR was also dependent on aging (*P* for trend < 0.001 and *P* for trend = 0.014, respectively). For subjects with Mt5178C genotype, the adjusted OR for reduced eGFR was significantly higher in those aged 50–59 and ≥ 60 years than in those aged < 50 years (adjusted OR = 3.955, 95% CI 1.821–8.591, *P* < 0.001 and adjusted OR = 7.961, 95% CI 3.261–19.43, *P* < 0.001, respectively). For subjects with Mt5178A genotype, the adjusted OR for reduced eGFR was significantly higher in those aged ≥ 60 years than in those aged < 50 years (adjusted OR = 3.667, 95% CI 1.355–9.921, *P* = 0.011).

**Table 3:** Odds ratios (ORs) and 95% confidence intervals (CIs) for reduced eGFR (< 90 mL/min/1.73 m^2^ or < 75 mL/min/1.73 m^2^) by Mt5178 C/A genotype and age group

Genotype and age group	Renal function		OR (95% CI)	Adjusted OR^†^ (95% CI)
	eGFR ≥ 90 mL/min/1.73 m^2^	eGFR < 90 mL/min/1.73 m^2^		
Mt5178C	*N* (%)	*N* (%)		
< 50 years	22 (34.9)	41 (65.1)	1 (reference)	1 (reference)
50–59 years	18 (14.8)	104 (85.2)	3.100 (1.509–6.370)**	3.086 (1.497–6.362)**
≥ 60 years	6 (9.7)	56 (90.3)	5.007 (1.863–13.45)**	4.206 (1.506–11.75)*
			*P* for trend < 0.001	*P* for trend = 0.001
Mt5178A	*N* (%)	*N* (%)		
< 50 years	16 (29.6)	38 (70.4)	1 (reference)	1 (reference)
50–59 years	18 (27.7)	47 (72.3)	1.099 (0.495–2.441)	1.053 (0.464–2.391)
≥ 60 years	5 (13.2)	33 (86.8)	2.779 (0.918–8.409)	2.070 (0.617–6.946)
			*P* for trend = 0.090	*P* for trend = 0.179
	eGFR ≥ 75 mL/min/1.73 m^2^	eGFR < 75 mL/min/1.73 m^2^		
Mt5178C	*N* (%)	*N* (%)		
< 50 years	53 (84.1)	10 (15.9)	1 (reference)	1 (reference)
50–59 years	70 (57.4)	52 (42.6)	3.937 (1.832–8.461)***	3.955 (1.821–8.591)***
≥ 60 years	24 (38.7)	38 (61.3)	8.391 (3.596–19.58)***	7.961 (3.261–19.43)***
			*P* for trend < 0.001	*P* for trend < 0.001
Mt5178A	*N* (%)	*N* (%)		
< 50 years	43 (79.6)	11 (20.4)	1 (reference)	1 (reference)
50–59 years	44 (67.7)	21 (32.3)	1.866 (0.804–4.330)	1.713 (0.712–4.126)
≥ 60 years	18 (47.4)	20 (52.6)	4.343 (1.733–10.89)**	3.667 (1.355–9.921)*
			*P* for trend = 0.002	*P* for trend = 0.014

†OR adjusted for smoking status and alcohol consumption

*P < 0.05, **P < 0.005, ***P < 0.001

## Discussion

In this exploratory cross-sectional study, we found that longevity-associated Mt5178 C/A polymorphism modifies the effect of aging on renal function. In simple linear regression analysis, a significant negative association between aging and eGFR was observed in both Mt5178C and Mt5178A genotypic men. Nevertheless, in multiple linear regression analysis, namely after adjustment, a significant effect of aging on reduced eGFR was observed only in men with Mt5178C genotype. In the case of reduced eGFR defined as < 75 mL/min/1.73 m^2^, reduced eGFR was dependent on aging in both Mt5178C and Mt5178A genotypic men. However, in the case of reduced eGFR defined as < 90 mL/min/1.73 m^2^, reduced eGFR was dependent on aging only in men with Mt5178C genotype.

There seems to be a genetic merit of Mt5178A genotype to lead longevity [9]. Individuals with Mt5178A genotype are reportedly resistant to adult-onset diseases, namely hypertension [10], diabetes [11], circulatory diseases [12–14], and Parkinson’s disease [28]. On the contrary, it is thought to be more arduous for individuals with Mt5178C genotype to realize longevity than those with Mt5178A genotype. The present study shows that physiological aging effect on reduced renal function is more evident in subjects with Mt5178C genotype than in those with Mt5178A genotype. Moreover, age is significantly and negatively associated with pulmonary function, namely forced expiratory volume in 1 s per forced vital capacity, only in men with Mt5178C genotype [17]. Considering both renal and pulmonary function, physiological aging seems to be more apparent in Mt5178C genotypic men than in Mt5178A genotypic men.

Based on the information of Mt5178 C/A polymorphism, lifestyle modification has the potential to improve physiological aging of renal function. We previously demonstrated that Mt5178 C/A polymorphism modulates the effect of alcohol consumption [15] or green tea consumption [16] on renal function. For Mt5178A genotypic men, habitual alcohol consumption reduces the risk of mildly decreased eGFR [15]. The risk of mildly decreased eGFR (< 90 mL/min/1.73 m^2^) was significantly lower in daily drinkers than in non-drinkers. Conversely, for Mt5178C genotypic men, green tea consumption increases the risk of mildly decreased eGFR [16]. The risk of mildly decreased eGFR (< 90 mL/min/1.73 m^2^) was significantly higher in subjects who consumed ≥ 6 cups of green tea per day than in those who consumed ≤ 1 cup of green tea per day. Previous molecular epidemiological studies on Mt5178 C/A polymorphism have reported gene-environment interactions on the risk of lifestyle-related diseases, namely hypertension [10], dyslipidemia [29], hyperuricemia [22], and liver injury [30]. Therefore, Mt5178 C/A genotyping may contribute to the establishment of individualized prevention not only for the physiological aging reduction of renal function but also for lifestyle-related diseases.

The present study demonstrated a significant positive association between aging and BUN levels in both Mt5178C and Mt5178A genotypic men. BUN levels, which have been utilized to evaluate renal function, are affected by nutrient intake [31]. Interaction between ND2-237 Leu/Met, namely Mt5178 C/A, polymorphism and β_3_-adrenergic receptor gene 64 (BAR3-64) Trp/Arg polymorphism on nutrient intake was reported [32]. BAR3-64 Trp/Trp genotype is the wild type, and BAR3-64 Trp/Arg or BAR3-64 Arg/Arg genotype is the variant type. Individuals with BAR3-64 wild type and Mt5178A genotype intake more carbohydrates and less animal protein than those with BAR3-64 wild type and Mt5178C genotype or those with BAR3-64 variant type, irrespective of Mt5178 C/A genotype. To verify BUN level through the abovementioned gene-gene interaction on eating behavior, a further genetic epidemiological investigation is required.

The underlying physiological mechanisms by which Mt5178 C/A, namely ND2-237 Leu/Met, polymorphism influences the effect of aging on kidney function remain unknown. NADH dehydrogenase, namely mitochondrial complex I, is recognized as the major site to release reactive oxygen species (ROS) in the mitochondria [33]. Extrapolation from an experimental animal model [34] to humans would suggest that ND2-237Met, deduced from Mt5178A, may suppress ROS production. This postulated suppression of ROS production may bring advantages for individuals with Mt5178A genotype in physiological aging of renal function. However, in contrast to conventional free radical theory in aging, the age-related increase in ROS production is recently thought to be relatively small and may not elucidate physiological changes in the aging process [4]. Therefore, further biophysical and biochemical investigations will be required.

This study evaluated only one single nucleotide polymorphism (SNP), namely Mt5178 C/A polymorphism. Longevity-associated Mt5178A genotype, recognized as mitochondrial haplogroup D, controversially contributes to the pathogenesis of early-onset end-stage renal disease [7]. Moreover, several SNPs in the mitochondrial displacement loop are potential predictors for CKD [8]. In addition to mitochondrial DNA SNPs, several SNPs of nuclear DNA are reportedly associated with CKD [35]. Recently, a trans-ancestry meta-analysis of genome-wide association studies of eGFR showed that 264 genetic loci, including 166 newly identified loci, were associated with kidney function [36]. Therefore, further genetic surveys will be necessary.

We use two definitions of reduced eGFR. Similar to our previous studies [15, 16] and other molecular epidemiological studies [24, 25], based on Kidney Disease Outcomes Quality Initiative CKD classification [26], reduced eGFR is considered to be < 90 mL/min/1.73 m^2^. A large-scale population-based survey showed that the prevalence of eGFR < 90 mL/min/1.73 m^2^ was 80.6% in Japanese men aged 30–69 years [37]. In this study, the prevalence of eGFR < 90 mL/min/1.73 m^2^ was also high, namely 82.4% in Mt5178C genotypic men and 75.2% in Mt5178A genotypic men. Moreover, based on an individual-level meta-analysis of 46 cohort studies [27], reduced eGFR is also considered to be < 75 mL/min/1.73 m^2^. Although, the lowest risk of mortality is at a GRF of ≥ 75 mL/min/1.73 m^2^ for age < 55 years, that is at GFR of 45–104 mL/min/1.73 m^2^ for age ≥ 65 years [1, 27]. In our study, the prevalence of moderately decreased eGFR of < 60 mL/min/1.73 m^2^, generally recognized as CKD, was very low in Mt5178C and Mt5178A genotypic men. Therefore, the validity of the two definitions of reduced eGFR adopted in this cross-sectional study is worthy of further deliberation.

As in other molecular epidemiological studies of renal function in the Japanese population [25, 35], the eGFR value was calculated using a three-variable Japanese equation [18]. Because this equation includes age as a predictor variable, a negative association between age and eGFR is naturally observed. All eGFR equations, utilized in many epidemiological studies, include age as a predictor variable [1]. Therefore, it is difficult to explain outcomes with eGFR across different age groups.

Several crucial limitations of this study should be discussed. First, the study sample was very small. Second, subjects comprised of only men. Third, we inspected only a single population. Precluding chance error in a molecular epidemiological study, it is obligatory for us to examine two or more independent datasets. Fourth, self-selection bias was not controlled. Individuals who visited the hospital for regular medical checkups were more health-conscious than those who did not receive health checkups. Fifth, although the cross-sectional study design can propose causal links, it cannot establish credible causality. Moreover, Chung et al. recommended longitudinal analysis to estimate and predict renal function decline rate with aging [38]. To overcome the abovementioned limitations, a prospective cohort study with a larger sample size including several populations is required. Finally, it was inferred from previous reports [39, 40] that lack of data on diet or physical activity was also a weakness of this study.

## Conclusions

This exploratory cross-sectional study suggests that Mt5178 C/A polymorphism may modify the effects of aging on renal function in male Japanese health checkup examinees. Aging effects on reduced renal function are thought to be more apparent in subjects with Mt5178C genotype than in those with Mt5178A genotype. In addition to resistance to adult-onset diseases [10–14, 28], from a physiological viewpoint, individuals with Mt5178A genotype realize longevity more effortlessly than those with Mt5178C genotype. Although the underlying physiological mechanisms have not been elucidated, molecular epidemiological studies on Mt5178 C/A polymorphism may contribute to the understanding of not only the aging kidney but also the physiological aging.

## Data Availability

The datasets used and/or analyzed during the current study are available from the corresponding author on reasonable request.

## Abbreviations

BAR3-64: β_3_-Adrenergic receptor gene 64
BMI: Body mass index
BUN: Blood urea nitrogen
CI: Confidence interval
CKD: Chronic kidney disease
eGFR: Estimated glomerular filtration rate
GFR: Glomerular filtration rate
Mt5178: Mitochondrial DNA 5178
ND2-237: NADH dehydrogenase subunit-2 237
OR: Odds ratio
ROS: Reactive oxygen species
SNP: Single nucleotide polymorphism
